# Conditional knockout of smooth muscle sodium calcium exchanger type‐1 lowers blood pressure and attenuates Angiotensin II‐salt hypertension

**DOI:** 10.14814/phy2.12273

**Published:** 2015-01-27

**Authors:** Youhua Wang, Ling Chen, Meng Li, Helen Cha, Takahiro Iwamoto, Jin Zhang

**Affiliations:** Department of Physiology, University of Maryland School of Medicine, Baltimore, Maryland, USA; Department of Medicine, University of Maryland School of Medicine, Baltimore, Maryland, USA; Department of Pharmacology, Faculty of Medicine, Fukuoka University, Fukuoka, Japan

**Keywords:** Angiotension II‐salt, blood pressure, myogenic response, phenylephrine, vascular smooth muscle

## Abstract

The functions of smooth muscle sodium calcium exchanger (NCX) in the vasculature are controversial and poorly understood. To determine the possible roles of NCX in the vascular phenotype and function, we developed a novel mouse model (SM‐NCX1 KO) in which the smooth muscle‐specific NCX type‐1 (NCX1) was conditionally knocked out using tamoxifen‐inducible Cre‐loxP recombination technique. SM‐NCX1 KO mice exhibit significantly lower blood pressure and attenuated angiotensin II (Ang II)‐salt‐induced hypertension (measured by radio telemetry and intra‐arterial catheterization). Isolated, pressurized mesenteric small resistance arteries from SM‐NCX1 KO mice, compared to control arteries, were characterized by the following: (1) ~90% reduced NCX1 protein expression; (2) impaired functional responses to (i) acute NCX inhibition by SEA0400 or SN‐6, (ii) NCX activation by low [Na^+^]_o_, and (iii) Na^+^ pump inhibition by ouabain; (3) attenuated myogenic reactivity; and (4) attenuated vasoconstrictor response to phenylephrine but not Ang II. These results provided direct evidence that arterial NCX1 normally mediates *net* Ca^2+^ influx that helps maintain basal vascular tone in small resistance arteries and blood pressure under physiological conditions. Importantly, NCX1 contributes to blood pressure elevation in Ang II‐salt hypertension, possibly by regulating *α*‐adrenergic receptor activation.

## Abstract (precis)

Smooth muscle‐specific Na/Ca exchanger type‐1 in adult mice was knocked out by the tamoxifen‐inducible Cre‐LoxP technique 3–5 weeks before experiments. This results in (1) attenuated myogenic response and attenuated vasoconstrictor response to alpha‐adrenoceptor activation in pressurized mesenteric small resistance arteries; and (2) lower baseline blood pressure and reduced angiotensin II‐salt‐induced hypertension.

## Introduction

The sodium‐calcium exchanger (NCX) is a bi‐directional regulator of intracellular [Ca^2+^] in the plasma membrane of most types of cells (Blaustein and Lederer 1999). NCX type‐1 (NCX1) is the predominant isoform in vascular smooth muscle (VSM) (Quednau et al. 1997; Iwamoto et al. 2004), and different splice variants of this isoform are ubiquitously expressed in other tissues. Determining the function of NCX in VSM, in particular whether it predominately extrudes or imports Ca^2+^, has proven to be particularly difficult in this system. Global NCX1 knockout in mice results in early embryonic death due to the lack of a beating heart (Koushik et al. 2001; Reuter et al. 2002), and such studies are therefore of limited utility. A particularly intriguing observation, however, is that mice that “overexpress” smooth muscle NCX1 (SM‐NCX1 TG) are hypertensive (Iwamoto et al. 2004; Zhang 2013). One possible explanation of this fact is that arterial NCX is normally providing *net* Ca^2+^ influx in the living animal (Zhang 2013). Only if NCX is providing net Ca^2+^ influx would more (i.e., overexpression) NCX provide more intracellular Ca^2+^ and consequent greater arterial constriction. In agreement with this, mice in which smooth muscle NCX1 was knocked out (Ren et al. 2010; Zhang et al. 2010; Zhao et al. 2011) during embryonic development are hypotensive, with reduced vascular contractility to myogenic‐ and vasoactive agonist‐stimulation in small resistance arteries. However, the permanent disruption of NCX1 from the embryonic stage may activate complicated compensatory mechanisms to offset the gain (transgenic “overexpression”) or loss (“knockout”) of function. For example, we observed attenuated PKC activation of L‐type Ca^2+^ channels (Ren et al. 2010) and renovascular responses to acute angiotensin II (Ang II) administration (Zhao et al. 2011) in NCX1 KO mice. To address this, we have now generated smooth muscle‐specific NCX1 knockout mice in a time‐dependent manner (conditional), using a tamoxifen‐inducible Cre‐LoxP recombination system. In this study, NCX1 gene in smooth muscle cells was deleted/inactivated 3–5 weeks before vascular function and BP were characterized. The goal of using these conditional, “loss‐of‐function” mice was to determine whether VSM NCX1: (1) mediates net Ca^2+^ flux under normal physiological conditions; (2) affects arterial contractility; and (3) regulates arterial BP under normal conditions and during salt‐dependent hypertension.

Our findings show that VSM NCX1 normally mediates *net* Ca^2+^ influx that contributes to the maintenance of myogenic vasoconstriction and regulation of BP. We also find that NCX1 is involved in agonist‐induced vasoconstriction in mouse mesenteric small resistance arteries, particularly in response to phenylephrine (PE). Information we obtained from the conditional SM‐NCX1 KO mouse model suggests that NCX in VSM plays an essential role under physiological conditions beyond its simple function as a Ca^2+^ transporter to maintain intracellular Ca^2+^ homeostasis. These results also help identify NCX1 as a potential molecular target for therapeutic intervention in cardiovascular diseases.

## Material and Methods

### Generation of conditional SM‐NCX1 KO mice

Transgenic mice having a tamoxifen‐inducible Cre gene driven by the smooth muscle specific‐myosin heavy chain promoter (smCre) were gifts from Dr. Steven Fisher (UMB). This mouse line did not show any Cre activity in the absence of tamoxifen. Mice homozygous for floxed NCX1 exon 11 (NCX1^Fx/Fx^) on C57BL/6 background, previously used for the generation of global smooth muscle‐specific NCX1 knockout mice (Ren et al. 2010; Zhang et al. 2010; Zhao et al. 2011), were crossed with smCre transgenic mice. This mating produced mice heterozygous for floxed NCX1 (NCX1^Fx/+^) and positive (smCre^+^) or negative (smCre^‐^) for tamoxifen inducible smCre. As smCre gene is located on the Y chromosome, smCre^+^ mice are all male. Offspring male mice (NCX1^Fx/+^/smCre^+^) were then crossed with female NCX1^Fx/Fx^ to generate offspring with four genotypes: NCX1^Fx/Fx^/smCre^+^, NCX1^Fx/Fx^/smCre^‐^, NCX1^Fx/+^/smCre^+^, and NCX1^Fx/+^/smCre^‐^. Offspring male NCX1^Fx/Fx^/Cre^+^ mice were then mated with female NCX1^Fx/Fx^ to generate NCX1^Fx/Fx^/smCre^+^ (male) or NCX1^Fx/Fx^/smCre^‐^ (female) mice. This mouse line was then stably maintained by crossing NCX1^Fx/Fx^/smCre^+^ (males) with NCX1^Fx/Fx^/smCre^‐^ (females) mice.

Only male NCX1^Fx/Fx^/smCre^+^ offspring, 12–15 weeks old, were used for experiments. In experimental NCX1^Fx/Fx^/smCre^+^ mice, Cre‐mediated excision and recombination of NCX1 exon 11 was turned on by tamoxifen injection (20 mg/ml in corn oil with 10% ethanol; ip/day × 5 consecutive days). The last day of tamoxifen injection was defined as day zero. After tamoxifen injection, mice (designated as SM‐NCX1 KO or KO in figures) appeared normal and were comparable to age‐matched NCX1^Fx/Fx^/smCre^+^ mice injected only with corn oil in 10% ethanol for five consecutive days (designated as controls or ctrl in figures). SM‐NCX1 KO mice had no significant behavior alteration.

### Ethical approval

All procedures involving mice complied with the standards stated in the National Institutes of Health Guide for the Care and Use of Laboratory Animals and were approved by the University of Maryland Animal Care and Use Committee.

### Genotyping

Floxed NCX1 primers were 5′‐AGGCATTCCAAAGGATGAGTGAAG‐3′ (forward) and 5′‐ATCCCAGTGGAGTTTGCTACCAGA‐3′ (reverse), yielding a WT band at 366 bp and the flox mutant band at 526 bp. The PCR protocol was 95°C for 5 min, then 10 cycles of 94°C for 15 sec, 65°C for 30 sec, and 72°C for 40 sec with a decrease of 1° per cycle in the annealing temperature, and then 30 cycles of 94°C for 15 sec, 55°C for 30 sec, and 72°C for 40 sec. The final elongation was 72°C for 7 min with a hold at 4°C.

smCre primers were SMWT1 (TGA CCC CAT CTC TTC ACT CC), SMWT2 (AAC TCC ACG ACC ACC TCA TC), and phCREAS1 (AGT CCC TCA CAT CCT CAG GTT). DNA from smCre^+^ mice yielded a band at ∼300 bp. The PCR protocol was 94°C for 5 minutes and then 94°C for 45 seconds, 62°C for 60 seconds, and 72°C for 60 seconds for 30 cycles, with a final elongation at 72°C for 10 minutes and a hold at 4°C.

### Telemetric arterial BP measurement

Mice were anesthetized with 1.5–2% of isoflurane supplemented with 100% O_2_. The right common carotid artery was exposed and ligated via an anterior neck midline incision. Radio telemetric BP sensors (DSI TA11PA‐C10, Data Science International, Minneapolis, MN) were used. The catheter of the BP sensor was inserted into a small hole proximal to the ligature, the tip was passed to the origin of the carotid at the aortic arch, and the catheter was fixed in place with a suture and the hole sealed with adhesive (Vetbond, 3M, St. Paul, MN). The transmitter was tunneled and then implanted to a subcutaneous pocket in the abdominal wall. Following 7–10 days of recovery from surgery, 24 h BPs were recorded for three consecutive days with DSI receivers and software for baseline measurement.

### Implantation of subcutaneous mini‐osmotic pumps for angiotensin II (Ang II) + high (6%) salt diet treatment

Mice with telemetric BP transducers implanted were anesthetized with 1.5% inhalational isoflurane (IsoFlo, Abbott Animal Health, Abbott Park, IL), weighed, and were then implanted subcutaneously an Alzet osmotic minipump (Model 1004, Durect Corporation, Cupertino, CA) filled with Ang II (350 ng kg^−1^ min^−1^). Mice were then treated with Ang II and high (6%) dietary salt intake for 4 weeks.

### Intracarotid artery BP measurement

Mice were anesthetized with isoflurane supplemented with 100% O_2_; core temperature was maintained at 37.5–38°C. The right common carotid artery was surgically isolated and cannulated with a 1.4 French Mikro‐tip pressure catheter (Millar Instruments, Houston, TX). BP was measured under 1.5% isoflurane anesthesia; data were calculated off‐line (BioPac System, Santa Barbara, CA). After the experiment, the animal was deeply anesthetized with isoflurane and euthanized by cervical dislocation.

### Isolated mesenteric small resistance artery preparation and functional study

Mice were euthanized with CO_2_ overdose followed by cervical dislocation, 3–5 weeks after tamoxifen/vehicle injection. Mesenteric small arteries were isolated and cannulated onto glass micropipettes and then mounted on a pressure myograph in organ bath using previously described method (Zhang et al. 2005, 2010). The artery in the bath was superfused with warmed physiological salt solution (PSS; 2 mL/min; 37 °C; 70 mmHg), which was bubbled with 5% O, 5% CO_2_, and balanced N_2_. Under no intraluminal flow conditions, arteries were allowed to develop spontaneous steady‐state myogenic tone (MT). Outside diameter was measured with edge‐detection technique using video microscopy and Labview software (National Instruments Corp., Austin, TX).

Following this equilibration period, myogenic‐, and agonist‐evoked vasoconstriction or vasodilation were measured as previously described (Zhang et al. 2005, 2010). Myogenic reactivity (MR) is expressed as diameter difference (Δ diameter) between active diameter in normal Ca^2+^‐containing PSS and passive diameter (PD, measured in [Ca^2+^]_o_‐free PSS at the conclusion of the experiment) at corresponding pressures (10–130 mmHg, 20 mmHg increment) as a percentage of the PD under 130 mmHg (PD_130_). The constrictor response to an agonist was measured by the extent of maximal contraction from MT to minimum diameter as a percentage of PD at 70 mmHg. Relaxation response was analyzed by measuring the extent of maximal relaxation, that is, increases in diameter, as a percentage relative to preconstriction, usually induced by 10 *μ*mol/L PE.

### Immunoblot analysis of inducible smooth muscle NCX1 deletion

Aorta, urinary bladder, heart and brain were dissected (on the same days as the functional studies on mesenteric arteries from the same animals were performed), minced, and homogenized in homogenization medium, and the membrane fractions were prepared to assay NCX1 protein expression. Tissue extracts were analyzed with specific monoclonal NCX1 antibody (R3F1, from K. D. Philipson, University of California, Los Angeles, CA). Band intensities were quantified with ID image analysis software (Eastman Kodak, Rochester, NY) and normalized with *β*‐actin to control for protein loading.

### Reagents and solutions

Artery dissection solution (mmol/L): 145 NaCl, 4.7 KCl, 1.2 MgSO_4_ · 7H_2_O, 2 MOPS, 0.02 EDTA, 1.2 NaH_2_PO_4_, 2 CaCl_2_ · 2H_2_O, 5 glucose; 2.0 pyruvate, 1% albumin (pH 7.4 at 5°C). PSS perfusion solution (mmol/L): 112 NaCl, 25.7 NaHCO_3_, 4.9 KCl, 2.5 CaCl_2_, 1.2 MgSO_4_ · 7H_2_O, 1.2 KH_2_PO_4_, 11.5 glucose, 10 HEPES (adjusted pH to 7.3–7.4 with NaOH). High (60 mmol/L) [K^+^]_o_ solution was made by replacing NaCl with equimolar KCl of normal PSS. Low (25.7 mmol/L) [Na^+^]_o_ solution was made by replacing NaCl with equimolar LiCl of normal PSS. Ca^2+^‐free solution was made by omitting Ca^2+^ and adding 0.5 mmol/L EGTA. Solutions were gassed with 5% O_2_, 5% CO_2_ and 90% N_2_.

Reagents and sources were as follows: angiotensin II (Ang II), endothelin‐1 (ET‐1), ouabain, PE, and U46619 (Sigma‐Aldrich, St. Louis, MO); SN‐6 (Tocris Bioscience, Minneapolis, MN); SEA0400 (2‐[4‐[(2,5‐difluorophenyl) methoxy]phenoxy]‐5‐ethoxyaniline, synthesized by Dr. T. Iwamoto, University of Fukuoka, Japan). Other reagents were reagent grade or the highest grade available. SN‐6 and SEA0400 were dissolved in DMSO; Others were dissolved in deionized water as stock solution. The final bath concentration of DMSO did not exceed 0.01% (v/v) and did not affect vascular function.

### Data analysis and statistics

The data are expressed as means ± SEM; *n* denotes number of arteries studied (1 artery per animal). Comparisons of data for single point between‐ and within‐group differences were made using Student's paired or unpaired *t* test as appropriate. One way or two way ANOVA was used when multiple points within‐ or between‐group differences were compared as indicated in figure legends. Differences were considered significant at *P *<**0.05.

## Results

### NCX1 protein expression is markedly reduced in SM‐NCX1 KO mouse arteries

**Figure 1.**
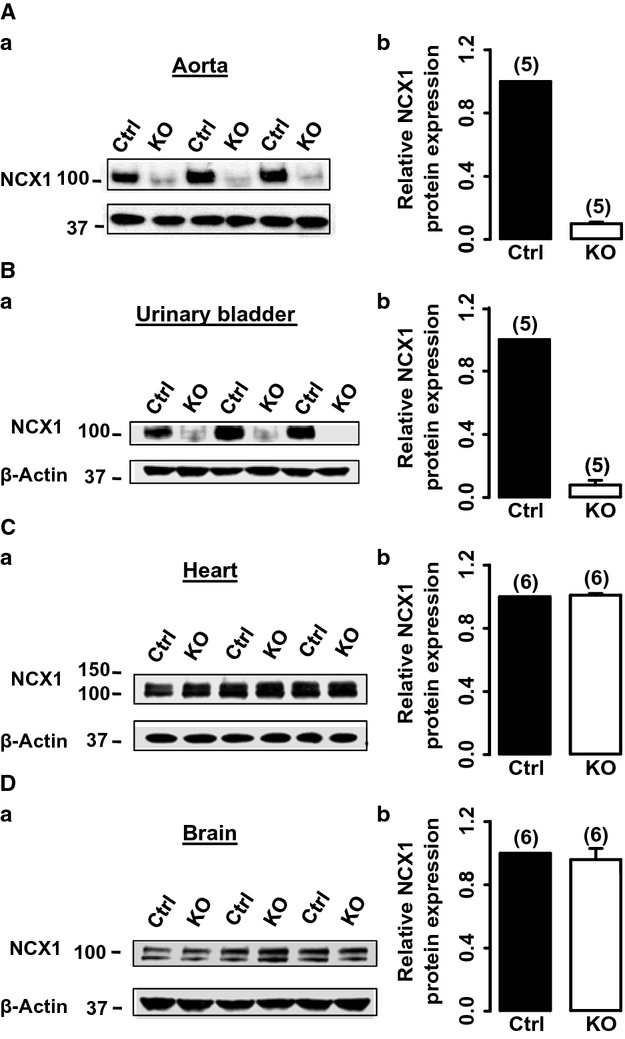
Confirmation of NCX1 protein expression knockout in arteries from tamoxifen‐induced conditional knockout of smooth‐muscle‐specific NCX1 (SM‐NCX1 KO) mice. Representative (a) and summarized (b) Western blot analysis of NCX1 protein in control and SM‐NCX1 KO mouse aorta (A), urinary bladder (B), heart (C), and brain (D). *P < 0.05. Numbers in parentheses indicate numbers of arteries included in each groups.

The efficiency of NCX1 knockout, specifically in VSM cells (VSMCs), was first determined by measuring the protein expression level of NCX1 in various tissues of NCX1^Flox/Flox^/smCre^+^ mice treated with vehicle (control) or tamoxifen (SM‐NCX1 KO). As illustrated in Fig. 1Aa, a distinct band at ~110 kDa was visible in all control arteries. NCX1 protein expression was then quantified in a total of six aorta samples from control and SM‐NCX1 KO mice, respectively. Data were normalized to the density of their respective positive control samples. NCX1 protein expression level was significantly reduced by ~90% in SM‐NCX1 KO arteries (Fig. 1Ab). NCX1 expression was similarly reduced in urinary bladders from SM‐NCX1 KO mice (Fig. 1B). In contrast, both hearts (Fig. 1C) and brains (Fig. 1D) from SM‐NCX1 KO mice expressed normal amount of NCX1 protein compared to those from control mice. In sum, these data demonstrate that the marked knockdown of NCX1 expression in SM‐NCX1 KO mice was restricted to smooth muscle cells.

### NCX1 function is markedly reduced in mesenteric small resistance arteries from SM‐NCX1 KO mice

**Figure 2.**
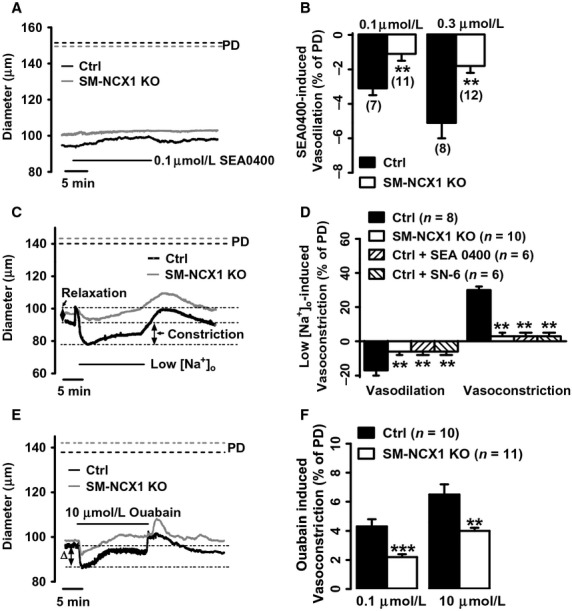
Functional confirmation of NCX1 knockout in mesenteric small resistance arteries from SM‐NCX1 KO mice. *A*, Representative recordings of vasodilation induced by 0.1 *μ*mol/L SEA0400 in a control (black line) and a SM‐NCX1 KO (gray line) mouse artery. Horizontal bar indicates time of exposure to SEA0400. Dashed line indicates passive diameter (PD, control, black; KO, gray), measured in [Ca^2+^]_o_‐free PSS at the conclusion of the experiments. *B*, Summary of vasodilation in response to 0.1 and 0.3 *μ*mol/L SEA0400 in arteries from control and SM‐NCX1 KO mice (numbers of arteries in parentheses). **P <**0.01. *C*, Representative experiments show the effects of lowering [Na^+^]_o_ from 137.7 to 25.7 mmol/L on the diameters of a control (black line) and a SM‐NCX1 KO (gray line) artery. *D*, Summary of effects of lowering [Na^+^]_o_ on the initial relaxation and delayed vasoconstriction in control, SM‐NCX1 KO arteries, and control arteries treated with 0.3 *μ*mol/L SEA0400 or 10 *μ*mol/L SN‐6. **P <**0.01 vs. control. *E*, Effects of 10 *μ*mol/L ouabain on the diameters of a representative control artery (black line) and a SM‐NCX1 KO (gray line) artery. *F*, summary of vasoconstrictions induced by ouabain (0.1 and 10 *μ*mol/L) in arteries from control and SM‐NCX1 KO mice. **P <**0.01; ***P <**0.001 vs. control.

Besides confirming the reduction in protein expression, we also assessed the loss‐of‐function of NCX1 in isolated, pressurized mesenteric small resistance arteries. Responses to specific NCX inhibitors, SEA0400 (Iwamoto et al. 2004; Zhang et al. 2010) or SN‐6 (Niu et al. 2007), activation of NCX1 by low [Na^+^]_o_, and inhibition of Na^+^ pumps by ouabain were tested. SEA0400 (0.1 *μ*mol/L) caused vasodilation in control arteries (Fig. 2A) as a result of decreased [Ca^2+^]_i_ (Zhang et al. 2010). In arteries from SM‐NCX1 KO mice, however, the effect of SEA0400 was significantly reduced (Fig. 2A). Similar effects were observed with 0.3 *μ*mol/L SEA0400 (Fig. 2B). SN‐6 (10 *μ*mol/L), another NCX1 inhibitor, caused vasodilation by 12 ± 2% in control arteries (*n *=**4), compared with only 3 ± 1% in SM‐NCX1 KO arteries (*n *=**6, *P *<**0.01). These results are consistent with our previous suggestion that arterial NCX1 mediates *net* Ca^2+^ influx in depolarized arteries, for example, pressurized arteries or arteries in vivo (Zhang et al. 2010; Zhang 2013).

As reported in conventional smooth muscle‐specific NCX1 KO mice (Zhang et al. 2010), exposure to low [Na^+^]_o_ is another common test of NCX function in small resistance arteries because the transmembrane Na^+^ electrochemical gradient is one of the factors that determine NCX functioning. Arteries were superfused with low (25.7 mmol/L) [Na^+^]_o_ solution for 15 min before normal (137.7 mmol/L) [Na^+^]_o_ was restored (Fig. 2C). This low [Na^+^]_o_ exposure resulted in an initial, transient vasodilation followed by a sustained vasoconstriction in control arteries (Fig. 2C). Preincubation of control arteries with 0.3 *μ*mol/L SEA0400 or 10 *μ*mol/L SN‐6 for 15 min reduced the dilation to low [Na^+^]_o_ solution by ~60% and the constriction by ~90% (Fig. 2D). Importantly, the magnitudes of both the transient vasodilation and the sustained vasoconstriction were similarly reduced in SM‐NCX1 KO arteries (Fig. 2C,D). Thus the effect of acute pharmacological inhibition on WT arteries is comparable with the effect of conditional genetic NCX1 ablation.

Additional evidence that arterial myocyte NCX1 activity is markedly reduced in SM‐NCX1 KO mice was obtained by exposing arteries to ouabain, the specific plasma membrane Na^+^ pump blocker. We have reported that ouabain raises cytosolic Ca^2+^ concentration and causes vasoconstriction in normal wild‐type arteries; these effects are markedly inhibited by SEA0400, suggesting that they result from increased NCX‐mediated Ca^2+^ entry (Iwamoto et al. 2004; Zhang et al. 2005, 2010). As illustrated in Fig. 2E, the exposure to ouabain (10 *μ*mol/L) caused a peak constriction followed by a plateau that sustained while ouabain was present in control arteries. The ability of ouabain to constrict arteries was significantly reduced in SM‐NCX1 KO arteries, manifested by a smaller peak and absent plateau. Similarly, the peak constrictor response to a lower dose (0.1 *μ*mol/L) ouabain, which blocks Na^+^ pumps with *α*2 isoforms only, was also significantly smaller in SM‐NCX1 KO (Fig. 2F) than in control arteries.

Thus, the absence of functional NCX1 in VSMCs was confirmed by the substantially attenuated vasoconstriction in responses to reduced [Na^+^]_o_, SEA0400, SN‐6, and ouabain, which was consistent with the readily detectable reduction in NCX1 protein expression in arteries (Fig. 1).

### Attenuated myogenic vasoconstriction in SM‐NCX1 KO mouse arteries

**Figure 3.**
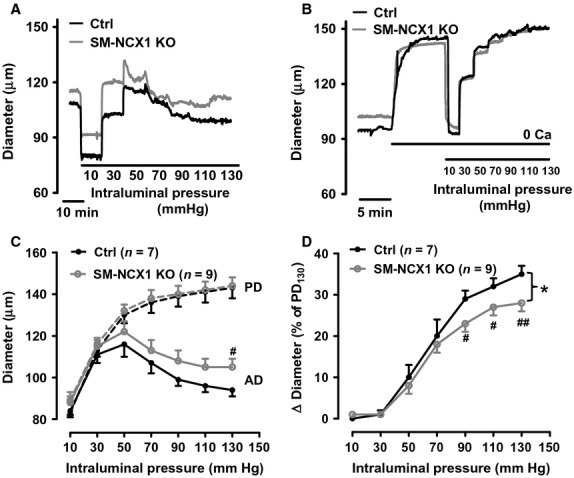
Effect of reduced NCX1 activity on myogenic reactivity (MR) in isolated, pressurized (70 mmHg) mesenteric small resistance arteries from control and SM‐NCX1 KO mice. Representative diameter changes in response to stepped increases in intraluminal pressure in arteries superfused with PSS (*A*) or [Ca^2+^]_o_‐free PSS (0Ca) (*B*) from control (black lines) and SM‐NCX1 KO (gray lines) mice. *C,* Mean steady‐state active diameter (AD, solid lines, superfused with PSS) and PD (dashed lines, measured in [Ca^2+^]_o_‐free PSS) of arteries in *A* and *B*. Control, black lines; SM‐NCX1 KO, gray lines. *D*, MR, expressed as differences between AD and PD (Δ diameter) at each pressures indicated on the abscissa as a percentage of PD at 130 mmHg (PD_130_) in arteries from control (black line) and SM‐NCX1 KO (gray line) mice. ^#^*P *<**0.05, ^##^*P *<**0.01 vs. control (Student's *t*‐test). *P <**0.05 (two way ANOVA).

Having confirmed the successful deletion of NCX1 protein expression and function, we then proceeded to assess the effect of NCX1 deletion on arterial contractility. When pressurized (70 mmHg) and in the presence of [Ca^2+^]_o_, mesenteric small resistance arteries develop spontaneous vasoconstriction, that is, MT. The basal active diameter, which was 111 ± 5 *μ*m or 19 ± 2% of PD (137 ± 4 *μ*m, *n* = 9) in arteries from SM‐NCX1 KO mice. These values were not significantly smaller than those in arteries from control mice (active diameter 108 ± 4 *μ*m; 21 ± 2% of PD, 136 ± 4 *μ*m, *n* = 7, *P *>**0.05). After MT was assessed, intraluminal pressure was reduced to 10 mmHg, then increased to 130 mmHg in 20 mmHg increments to compare active vasoconstriction (Fig. 3A) and passive dilations in the absence of [Ca^2+^]_o_ (Fig. 3B). When active diameter (AD) and passive diameter (PD) were plotted as a function of intraluminal pressure, arteries from SM‐NCX1 KO mice exhibited significantly reduced myogenic (active) vasoconstriction than did arteries from control mice (Fig. 3C). Thus, MR curve was significantly shifted downwards in arteries from SM‐NCX1 KO mice (Fig. 3D).

### Effect of knockout of smooth muscle NCX1 on agonist‐induced vasoconstriction

**Figure 4.**
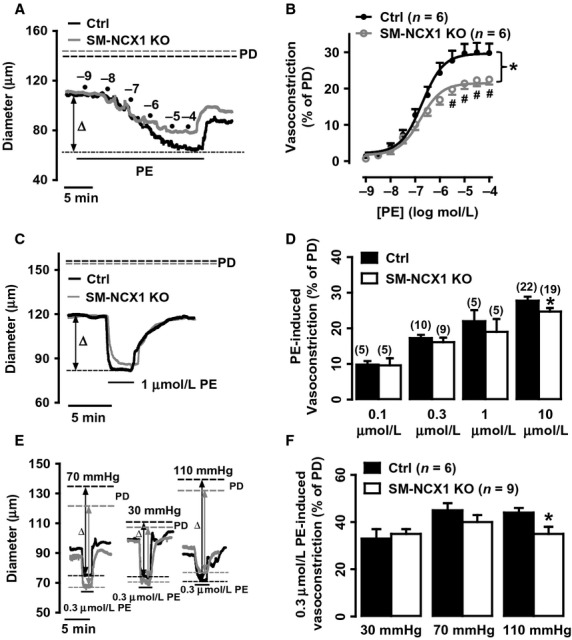
Attenuated phenyleprine (PE)‐induced vasoconstriction in arteries from SM‐NCX1 KO mice. *A*, Representative experiments show the dose‐dependent (0.001–100 *μ*mol/L) vasoconstrictor effect of PE in a control (black line) and a SM‐NCX1 KO (gray line) artery. *B*, Summarized data showing the average diameter changes in response to cumulative PE application in arteries from control and SM‐NCX1 KO mice. *C*, Representative experiments show the effect of 1 *μ*mol/L PE in a control (black line) and a SM‐NCX1 KO (gray line) artery. *D*, Summary of vasoconstrictions in response to single dose application of PE in mesenteric small resistance arteries from control and SM‐NCX1 KO mice. *E*, Representative experiments show the effect of increasing or lowering intraluminal pressure on 0.3 *μ*mol/L PE‐induced vasoconstriction in a control artery (black line) and a SM‐NCX1 KO artery (gray line). *F*, Summary of vasoconstrictions in response to 0.3 *μ*mol/L PE under 30, 70, and 110 mmHg intraluminal pressures in mesenteric small resistance arteries from control and SM‐NCX1 KO mice. *P <**0.05.

Contractile responses to vasopressor agents in small resistance arteries provide useful information to evaluate possible changes in peripheral resistance in hypertension. As increased sympathetic nerve activity has been found in many hypertension animals (Osborn et al. 2011; Wang et al. 2013) and human patients with hypertension (Schlaich et al. 2004), we therefore first compared vasoconstrictions induced by a sympathomimetic‐based vasopressor agent, PE. Cumulatively administered PE (0.001 ‐ 100 *μ*mol/L) caused dose‐dependent vasoconstriction in a control artery, which was attenuated in a SM‐NCX1 KO artery (Fig. 4A). Summary data show that PE at > 3 *μ*mol/L caused significantly reduced constriction in SM‐NCX1 KO arteries, but the concentration required for half‐maximal constriction was not significantly different between control (0.19 *μ*mol/L) and SM‐NCX1 KO arteries (0.16 *μ*mol/L) (Fig. 4B). To exclude possible desensitization due to prolonged cumulative administration, maximal vasoconstriction induced by a single dose of various concentrations (0.1, 1, and 10 *μ*mol/L) of PE was compared. As illustrated in Fig. 4C, 1 *μ*mol/L PE caused slightly, but not significantly, smaller contraction between control and SM‐NCX1 KO arteries, similar to the effect observed during cumulative application. Consistently, the maximal contraction in response to high dose (10 *μ*mol/L) was still significantly reduced in SM‐NCX1 KO arteries (Fig. 4D). Similar results were found during prolonged (15–20 min) PE administration (data not shown). As *α*‐adrenoceptor‐mediated Ca^2+^ signaling and vasoconstriction are affected by intraluminal pressure and MT (Zacharia et al. 2007), PE‐induced vasoconstriction was also compared in arteries that developed MT but under lower (30 mmHg) or higher (110 mmHg) intraluminal pressures than the ideal (70 mmHg) intraluminal pressure. Interestingly, when intraluminal pressure was elevated, the vasoconstriction to PE was attenuated in control arteries, but even more so in SM‐NCX1 KO arteries, such that at even lower concentration (e.g., 0.3 *μ*mol/L), PE‐evoked vasoconstriction was significantly smaller in SM‐NCX1 KO arteries (Fig. 4E,F).

**Figure 5.**
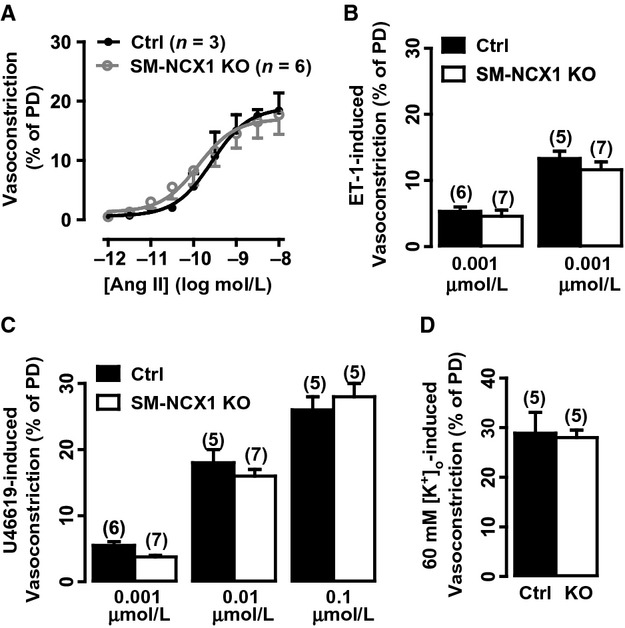
Agonist‐induced vasoconstrictions in arteries from control and SM‐NCX1 KO mice. *A*, Summarized data showing the average diameter changes in response to cumulative Ang II application in arteries from control and SM‐NCX1 KO mice. Summarized data showing the average diameter changes in response to single dose of ET‐1 (*B*), U46619 (*C*), and high [K^+^]_o_ solution (*D*), applied to evoke vasoconstrictions in mesenteric small resistance arteries from control and SM‐NCX1 KO mice. Numbers in parentheses indicate numbers of arteries included in each groups.

The contractile properties of the SM‐NCX1 KO arteries in response to other agonists were also determined. Although a potent vasoconstrictor and important in hypertension, Ang II, in contrast to PE, caused similar dose (0.000001–0.01 *μ*mol/L)‐dependent vasoconstriction in SM‐NCX1 KO and control arteries (Fig. 5A). Also, there were no significant differences between control and SM‐NCX1 KO arteries in vasoconstrictions induced by ET‐1 (0.001 and 0.01 *μ*mol/L, Fig. 5B), U46199 (0.001, 0.01, 0.1 *μ*mol/L, Fig. 5C), or high [K^+^]_o_ (60 mmol/L) solution (Fig. 5D).

### Significantly lower BP and attenuated response to Ang II‐salt in SM‐NCX1 KO mice

The attenuated MT, MR, and PE‐induced vasoconstriction led us to examine BP in SM‐NCX1 KO mice. Mean arterial BP (MAP), measured with an intracarotid artery catheter in mice under light isoflurane anesthesia, was 98 ± 4 mmHg (*n *=**11) in control mice, and was significantly decreased in SM‐NCX1 KO mice (80 ± 2 mmHg, *n *=**14; *P *<**0.001. Table 1). Systolic (SBP) and diastolic BP (DBP) were consistently decreased. In comparison, heart rate (HR) of SM‐NCX1 KO mice was not significantly different from that of controls (Table 1).

**Table 1.** Hemodynamic parameters measured by intracarotid artery catheter in anesthetized control and SM‐NCX1 KO mice. There are significant differences in SBP, DBP, and MAP between control and SM‐NCX1 KO mice. There are no statistical differences in HR or body weight (BW) between control and SM‐NCX1 KO mice

	SBP (mmHg)	DBP (mmHg)	MBP (mmHg)	HR (bpm)	BW (g)	*n*
Ctrl	117 ± 4	84 ± 3	98 ± 4	527 ± 15	36 ± 2	11
KO	95 ± 3*	68 ± 2*	80 ± 2*	521 ± 15	33 ± 2	14

* *P *<**0.001.

**Figure 6.**
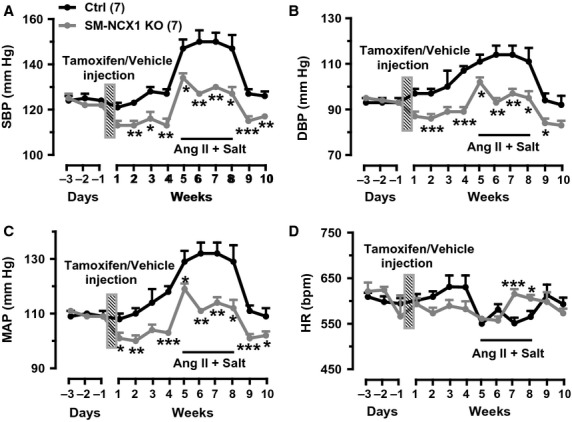
Arterial blood pressure and heart rate in control and SM‐NCX1 KO mice in response to chronic Ang II administration plus high dietary salt intake. Averaged 24 h systolic blood pressure (SBP,* A*), diastolic BP (DBP,* B*), mean arterial blood pressure (MAP,* C*) and heart rate (HR,* D*) measured by radio telemetry on the indicated time before and after tamoxifen injection, and in response to Ang II administration in mice consuming a high salt diet (6%). Shaded rectangle bars indicate time of tamoxifen (SM‐NCX1 KO) or vehicle (control) injection. *P <**0.05; **P <**0.01; ***P <**0.001; Student's *t* test. *n *=**7 control and 7 SM‐NCX1 KO mice.

When measured by radiotelemetry in conscious, free moving mice, before tamoxifen injection, the baseline SBP, DBP, MAP, or HR throughout each 24 h period over three consecutive days were not significantly different between control and SM‐NCX1 KO mice (Fig. 6A–D). The average MAP over 3 days was 110 ± 1 mmHg (*n *=**7) in SM‐NCX1 KO mice, which was not different from that in control mice (109 ± 0.3 mmHg, *n *=**7, *P *>**0.05). After 5 days of consecutive tamoxifen injection, BP was recorded continuously for 24 h every other day for 4 weeks before mice were treated with Ang II plus salt. Tamoxifen injection caused baseline MAP to drop at week 1 (Fig. 6C). Average baseline MAP over 4 weeks after tamoxifen injection was significantly lower in SM‐NCX1 KO mice (102 ± 1 mmHg vs. 112 ± 2 mmHg in controls) (Fig. 6C). Similar trends were observed in SBP (Fig. 6A) and DBP (Fig. 6B) with tamoxifen injection. HR, in contrast, was not significantly affected by tamoxifen or vehicle injection (Fig. 6D).

Subcutaneous administration of Ang II plus a high salt (6%) diet induces salt‐sensitive hypertension in normal mice resulting from activation of central neural mechanisms that increase sympathetic nerve activity (Osborn et al. 2011; Blaustein et al. 2012). In control mice, average MAP throughout the 4 weeks (two periods of 24 h‐recording each week) of Ang II‐salt treatment was elevated to 130 ± 1 mmHg. In contrast, the average MAP during Ang II‐salt treatment in SM‐NCX1 KO was 114 ± 2 mmHg. Thus, the BP response was significantly attenuated (*P *<**0.001) in SM‐NCX1 KO mice during Ang II infusion (Fig. 6C). This effect was not a result of decreased HR, because HR was even significantly higher in SM‐NCX1 KO mice during Ang II‐salt treatment (Fig. 6D).

## Discussion

In this study, we sought to determine the role of VSM NCX1 in small resistance arteries by characterizing the vascular phenotypic and functional changes in response to conditional knockout of smooth muscle NCX1. We measured basal myogenic response, agonist‐induced vasoconstriction in isolated, pressurized mesenteric small arteries (PD is ~140 *μ*m at 70 mmHg), and BP in both awake and anesthetized mice. The observed changes compared to those in control mouse arteries are supposed to be mediated directly by NCX1 in this time‐dependent (viz, *conditional*), tissue‐specific “loss‐of‐function” preparation, in order to exclude possible compensatory mechanisms that are commonly invoked in conventional gene knockout technique. Compared to our previous work on conventional smooth muscle‐specific NCX1 knockout mouse model (Zhang et al. 2010), the conditional KO not only confirmed the phenotypic changes including reduced baseline MAP and correlated attenuated MR, attenuated PE‐induced vasoconstrictor response, but also provided direct evidence and mechanisms for these observations. Furthermore, the significantly attenuated Ang II‐salt‐induced hypertension in SM‐NCX1 KO mice demonstrates a crucial role of NCX1 in the regulation of BP in hypertension and suggest that NCX1 is also importantly involved in pathological conditions.

NCX is expressed ubiquitously in vasculature, including endothelial cells (Teubl et al. 1999), VSMCs (Iwamoto et al. 2004; Raina et al. 2008), and possibly peri‐vascular nerve ending cells (Motley et al. 1993). In VSMCs, NCX1 is the predominant isoform (Quednau et al. 1997; Iwamoto et al. 2004). The primary role of NCX1 in normal healthy cells has been widely regarded as a Ca^2+^ extrusion system, which is why the Ca^2+^ efflux mode is referred to as the “forward” mode of NCX (Noble and Blaustein 2007). In spite of this broad agreement on the NCX function in many cell types (e.g., heart and neurons), the range of functions of NCX1 in VSMCs has been controversial and incomplete, even as to which direction of Ca^2+^ flux NCX1 mediates under normal physiological conditions (Horiguchi et al. 2001; Rebolledo et al. 2006; Akolkar et al. 2012). Although it seems likely that in small arteries in living animals, NCX1 can operate in either “Ca^2+^ influx” or “efflux” mode, depending on the prevailing conditions (Zhang 2013), our recent evidence supports the idea that in the basal state of the animal, the predominant mode of NCX1 is “Ca^2+^ influx” mode. This has been reflected in the current and previous studies (Zhang et al. 2010) by the reduced MT, MR, attenuated vasoconstrictor responsiveness, significantly lowered BP, and attenuated BP response to Ang II‐salt hypertension in SM‐NCX1 KO mice. Consistently, enhanced small arterial response to ouabain stimulation, and hypersensitivity to salt‐induced BP elevation were observed in smooth muscle‐specific NCX1 overexpressing mice (Iwamoto et al. 2004). Importantly, results obtained in these genetically engineered mice in which NCX1 protein expression and function was decreased specifically in smooth muscle cells have been consistent with those obtained in normal mouse arteries in which NCX function was blocked by either a specific inhibitor, SEA0400, or by antisense oligonucleotide knockdown of NCX (Raina et al. 2008). On the basis of these highly consistent results, we have suggested that VSM NCX1 normally mediates *net* Ca^2+^ influx that is required for the maintenance of basal arterial tone, and agonist‐induced vasoconstriction in small resistance arteries and, consequently BP, under basal physiological conditions.

MT, the basal level of arterial tone, on top of which arteries respond to neurohumoral stimuli, is important in virtually all aspects of vascular function under both physiological and pathological conditions (Davis and Hill 1999). In hypertension, MT is likely a component of the initial auto‐regulatory response to tissue overperfusion when BP rises as a consequence of volume expansion. Our group has shown that MT is positively correlated with BP change in several animal hypertension models (Iwamoto et al. 2004; Zhang et al. 2005, 2010; Linde et al. 2012). Here, we have demonstrated the direct contribution of NCX1 to MT: isolated, pressurized mesenteric small arteries from conditional SM‐NCX1 KO mice exhibit significantly reduced MR (Fig. 3). Furthermore, acute inhibition of NCX by SEA0400 reduces MT in control [Fig. 2, also see (Zhang et al. 2005, 2010)], but not in SM‐NCX1 KO mouse arteries (Fig. 2). Vasoconstriction induced by lowering [Na^+^]_o_, resulting from decreased Ca^2+^ extrusion and/or increased Ca^2+^ entry via NCX, is remarkably reduced in SM‐NCX1 KO arteries (Fig. 2). These impaired functions reflect the reduced NCX protein expression in mesenteric arteries (Fig. 1). Conversely, SM‐NCX1 TG arteries have significantly enhanced MT (T. Iwamoto & J. Zhang, unpubl. data). These results are in agreement with observations by our previous work (Zhang et al. 2010) and by others (Raina et al. 2008; Kashihara et al. 2009). The data obtained from conditional SM‐NCX1 KO mice now has provided direct evidence and confirmed our hypothesis that arterial NCX1 normally mediates *net* Ca^2+^ entry for the development or maintenance of MT.

Although the data from this study support a role for NCX1 in mediating “net Ca^2+^ entry”, direct measurement and comparison of cytosolic [Ca^2+^] between control and SM NCX1‐KO arteries would represent an important “mechanistic” element for an extension of this study. However, measurements of Ca^2+^ concentration in intact arteries cannot be performed reliably with currently available Ca^2+^ indicators: fluo‐4 or fura‐2. Fluo‐4 is not ratio‐metric, therefore it cannot be used to determine differences between different arteries. Although fura‐2 has been used in some cases, the absolute fura‐2 calibration is plagued by numerous errors, including “protein binding, extracellular leakage, and loading of fura‐2 into organelles” (Rembold and Chen 1998). In our own experience, a large (and variable) tissue background and out‐of‐focus fluorescence, variable dye loading from artery to artery, and inability to obtain true R_Max_ and R_Min_ values in intact arteries, are other major problems that preclude meaningful comparison of results in different arteries. In contrast, we have developed a method recently for longitudinal quantitative measurements of [Ca^2+^] in arteries of optical biosensor mice (Mauban et al. 2013; Wang et al. 2013; Fairfax et al. 2014). This method avoids the problems associated with fura‐2. However, the use of biosensor mice in this study would require hybridization and back‐crossing of the biosensor mice with the NCX‐altered mice. This was well beyond the scope of this study because of the time that would have been required. Similarly, transfection of isolated arteries with the biosensor DNA (plasmid), which might be useful in theory, has proved difficult and not successful often enough to be useful.

In hypertension, there has long been direct evidence of structural remodeling of arteries that causes narrowed lumens. Experimentally, eutrophic remodeling (Martinez‐Lemus et al. 2009) under strong vasoconstrictor action can occur quite rapidly (hours). Such structural changes persist in living arteries if studied immediately ex vivo, and are evident as a decrease in PD (as measured in Ca^2+^ free conditions (Korsgaard et al. 1993). We also evaluated the structure of mesenteric small resistance arteries and possible vascular remodeling by measuring the internal and external diameters during maximal relaxations to [Ca^2+^]_o_‐free PSS plus EGTA. There were no differences between control and SM‐NCX1 KO mouse arteries in maximal dilation, wall thickness, wall to lumen ratio, or cross‐sectional area of the vessel wall at all levels of intraluminal pressure (Data not shown), suggesting that structural remodeling is not obvious in KO arteries. Thus, whether structural remodeling occurs in hypotension, as occurred here, is not known.

The role of VSM NCX1 in regulating agonist‐induced contractions has been reported, for example, “Ca^2+^ entry” mode of NCX has been shown to be activated during alpha‐adrenergic receptor (Khoyi et al. 1991; Fameli et al. 2007; Dai et al. 2010) and purinergic receptor (Poburko et al. 2007; Syyong et al. 2007) stimulation in VSMCs, and during ET‐1‐induced contraction in rat vena cava, but not aorta (Tykocki et al. 2012). These typical vasoconstrictions induced by activation of G protein‐coupled receptors is thought to be initiated by IP_3_‐sensitive SR Ca^2+^ release and maintained by Ca^2+^ entry from extracellular fluid (Poburko et al. 2007; Syyong et al. 2007). The two products of G protein‐coupled receptor activation, IP_3_ and DAG, are both indirectly linked to VSM NCX1. DAG activates the Na^+^ permeable TRPC6, leading to local Na^+^ accumulation and Ca^2+^ influx via NCX (Poburko et al. 2007; Syyong et al. 2007), which refills the sarco‐/endo‐plasmic reticulum Ca^2+^ pool via SERCA, a process that is aided by the spatial co‐localization of VSM NCX and SERCA (Davis et al. 2009). IP_3_ triggers sarco‐/endo‐plasmic reticulum Ca^2+^ release and the initial “phasic” vasoconstriction, which increases sarco‐/endo‐plasmic reticulum ‐dependent Ca^2+^ waves that activate Ca^2+^/calmodulin‐dependent myosin light chain kinase. Interestingly, deletion of smooth muscle NCX1 only affects vasoconstrictor responses to PE (Fig. 4), but not to the other G‐protein coupled receptor agonists tested here (Fig. 5). NCX is thought to be particularly involved in *α*1‐adrenoceptor‐mediated responses to PE (Dai et al. 2010), but its involvement in response to activation of other G‐protein coupled receptor is less well known. It is possible that ET‐1, U46619 and other agonists, which have different physiological effects, and which activate different G‐protein coupled receptor, do not activate the same signaling pathways that result in Ca^2+^ entry via NCX. The mechanisms responsible for the attenuated PE‐induced vasoconstriction are uncertain. Preliminary data imply that the mobilization of stored Ca^2+^ is not impaired in arteries from NCX1 KO mice because high PE‐induced vasoconstriction in Ca^2+^‐free PSS is not attenuated (data not shown). One possible explanation is that NCX1 is more functionally involved in α‐adrenoceptor activation as a result of sensitization to the ongoing sympathetic nerve activity.

In line with these findings, our studies on both conventional (Zhang et al. 2010) and conditional smooth‐muscle‐specific NCX1 knockout mice have shown reduced vasoconstrictor response to PE (Fig. 4) in mesenteric blood vessels. As we have noted that abolition of NCX1 leads to the compensatory decreased activation in L‐type voltage‐gated calcium channel in conventional KO mice (Zhang et al. 2010), in contrast to this finding, in this study, high [K^+^]_o_‐exposure resulted in similar vasoconstrictions in control and SM‐NCX1 KO arteries. This indicates that the maximal Ca^2+^ entry through L‐type voltage‐gated calcium channels is possibly normal in SM‐NCX1 KO arteries because the high [K^+^]_o_‐evoked vasoconstriction is abolished by the L‐type voltage‐gated calcium channels blocker, nifedipine [data not shown in this study, see (Zhang et al. 2007, 2010)]. Therefore, unlike in the conventional KO model, in which the observed attenuation of PE‐induced vasoconstriction might be attributed to less Ca^2+^ entry through L‐type voltage‐gated calcium channels, here, the results indicate that the attenuated PE‐induced vasoconstriction was a result of NCX1 functional deletion, that is, NCX1 contributes directly to PE response. Thus, by comparing the difference between the conventional (long‐term) and conditional (short‐term) ablation of NCX1 function, one can see the great advantage of using conditional knockout technique.

Vascular tone, as discussed above, is a primary determinant of peripheral resistance and BP (Davis and Hill 1999). Regulation of vascular tone/resistance involves multiple mediators under both normal and disease conditions, including those from VSM, perivascular nerve endings, endothelium, and circulating blood. In isolated arterial preparations (Figs. 2–5), arterial functional and structural changes can be assessed independently from neural or hormonal factors present in living animal. This facilitates uncovering alterations, such as attenuated MT, that are usually not possible to detect under in vivo conditions. Such alterations, of course, ultimately have to be verified in vivo under the challenge of more complex situation. In this study, the sudden drop of BP to a significantly lower level in the first week after tamoxifen injection (Fig. 6A–C) might be the in vivo correlation of the attenuated MT due to VSM NCX1 deletion [successful deletion of NCX1 protein expression also occurs as early as 1 week after tamoxifen exposure (data not shown)], in the isolated mesenteric small resistance artery, because both MT and BP (Fig. 6) are normal before tamoxifen injection.

Ang II‐salt hypertension is a typical neurogenic salt‐sensitive hypertension model (Osborn et al. 2011). In view of the pathogenesis of Ang II‐salt hypertension, one of the key features is the increased central sympathetic outflow, which results in augmented sympathetic activation of the splanchnic vascular bed, thus, increases vascular resistance and BP (Osborn et al. 2011). Just as hypertension is a multifactorial disease that involves multiple organs (Blaustein et al. 2012), elevated BP in Ang II‐salt model also can result from abnormalities of any factors or organs. In this study, increased sympathetic flow as a natural outcome of the central neural mechanisms in response to Ang II‐salt is still normal in SM‐NCX1 KO mice as manifested by the significantly increased HR at weeks 3 and 4 during Ang II‐salt treatment (Fig. 6D). In addition, SM NCX1‐KO mouse arteries respond normally to acutely administered Ang II (Fig. 5A), and only exhibit reduced hypertensive response to chronically infused Ang II (‐salt) (Fig. 6), suggesting this is not due to hypersensitivity of blood vessels to Ang II. These evidence are consistent with the central neural mechanisms of “Ang II‐salt sympathetic signature” (Osborn et al. 2011), but also suggest defects of certain downstream vascular components, which in our study is the VSM NCX1, that mediates the activation of sympathetic drive and the regulation of BP in salt‐sensitive hypertension. These defects, such as the reduced vascular sensitivity to *α*‐adrenergic stimulation, especially under higher intraluminal pressure (Fig. 4), as a result of NCX1 deletion, attenuated the augmented influence of sympathetic outflow on vascular resistance and BP, hence significantly reduced the outcome. Finally, the data in this study indicate that NCX1 is critically involved in the regulation of arterial BP. We might expect also that the reduction in MR (Fig. 3) might also result in diminished blood flow regulation and possible end‐organ damage, although these phenomena were not measured in this study.

In sum, the conditional, inducible smooth muscle‐specific NCX1 knockout mouse model generated in this study allows us to investigate the role of smooth muscle NCX1 directly, without the possible interference of compensatory or secondary mechanisms that can arise in conventional (germline) knockout mouse models. Germ‐line smooth muscle‐specific NCX1 knockout also produced animals with significantly attenuated arterial MT and PE‐response, as well as decreased BP (Zhang et al. 2010). However, attenuated activation of L‐type voltage‐gated Ca current, or secondary complications consequent to germ‐line deletion, for example, the activation of Cre during gametogenesis yielding heterozygous offspring, have been noticed (Zhang et al. 2010). These phenomena confound the interpretation of attenuation of vascular response and reduced BP. In contrast, the results we described here strengthen the view that NCX1, by itself, plays a direct and important role in the regulation of vascular function and BP, both under normal condition and in response to Ang II + salt‐induced hypertension.

## Acknowledgments

We thank Drs. W. Gil Wier and Mordecai P. Blaustein (University of Maryland) for very helpful discussion and comments on the manuscript. We also thank Dr. Steven A. Fisher (Univ. of Maryland) for gifts of smCre mice and Dr. Kenneth D. Philipson (UCLA) for NCX1^Fx/Fx^ mice. We greatly appreciate Dr. Blaustein's effort in putting together these two mouse lines to start the conditional knockout colony.

## Conflict of Interest

No conflicts of interest, financial or otherwise, are declared by the authors.
